# Relationships Between Cull Beef Cow Characteristics, Finishing Practices and Meat Quality Traits of *Longissimus thoracis* and *Rectus abdominis*

**DOI:** 10.3390/foods8040141

**Published:** 2019-04-25

**Authors:** Sébastien Couvreur, Guillain Le Bec, Didier Micol, Brigitte Picard

**Affiliations:** 1 Unité de Recherche sur les Systèmes d’Elevage, Ecole Supérieure d’Agricultures, Université Bretagne-Loire, 55, rue Rabelais, BP 30748, F-49007 Angers, France; g.lebec@groupe-esa.com; 2 Université Clermont Auvergne, INRA, VetAgro Sup, UMR Herbivores, F-63122 Saint-Genès-Champanelle, France; didier.micol@inra.fr (D.M.); brigitte.picard@inra.fr (B.P.)

**Keywords:** suckling cattle, cull cow, meat quality, finishing practices, farm survey

## Abstract

The aim of study was to investigate the relationships between the characteristics of cull beef cows in the Rouge des Prés breed, finishing practices and physicochemical characteristics and sensory traits of *Longissimus thoracis* (LT) and *Rectus abdominis* (RA) muscles from 111 cows. On the basis of our surveys, which qualify at cow level the animal characteristics and finishing diet, clusters of cull cows and finishing practices are created and their effects tested on LT and RA meat quality. Old and heavy cows with good suckling ability (95 months, 466 kg and 7.1/10) are characterized by LT with larger fibers, and higher intramuscular fat content and fat-to-muscle ratio. Young and heavy cows with low suckling ability (54 months, 474 kg and 4.4/10) are characterized by LT and RA with lower MyHC IIx and higher MyHC IIa and MyHC I proportions. MyHC IIx and IIa proportions are lower and a* and b* color indices higher when cows are finished on pasture, probably related to grass diet and physical activity. The fat-to-muscle ratio is higher without any effect on the intramuscular fat content when cows are finished over a short period (107 days) with a high level of concentrate (9.7 kg/day). The opposite effect is observed over a long period (142 days) with a low level of concentrate (5.8 kg/day), confirming the interaction effect between finishing duration and amount of energy concentrate on the allotment of adipose tissue deposit.

## 1. Introduction

In France, more than 50% of the beef meat produced and consumed comes from cull dairy and beef cows. There is a high diversity of cows in terms of breed, age, and diets. Throughout their life, cows are fed with different diets (maize or grass silage, pasture, and hay associated with energy and protein supplementations) which led to different growth curves and fat deposition dynamics [1]. Before slaughtering, they can be fed with a finishing diet for several weeks which influences the muscle and fat contents of the carcass. This can induce a wide variability in carcass and meat quality traits such as tenderness, juiciness, or flavor [2,3]. Many experiments dealing with tenderness have shown that age can have a negative effect on tenderness from 24 months to more than 60 months [4,5]. Moreover, compensatory growth, high energy level, growth rates, and duration of finishing periods can have positive effects on tenderness that vary according to animal type (steer, heifer, young bull, or cull cow) and muscle [3]. Finally, morphological animal type has few effects on tenderness [6]. The effects of diet and animal type on flavor and juiciness have been assessed in a few studies. Duarte et al. [7] have shown, in Nellore cattle, that juiciness and flavor are less affected by the finishing diet and have a negative correlation to growth rate. Finishing diets leading to high fat deposition (and low growth rate), such as a long fattening period with a low energy level diet, may increase juiciness and flavor intensity [8,9].

Nevertheless, most studies dealing with the effects of diet and animal type on meat quality have mainly been conducted on young animals such as heifers, young bulls, and steers. In cull cows, few experiments have been conducted dealing with the effect of the breed [4,5,6], finishing diet [9,10], and age [5]. The main part of these studies has been conducted on cull dairy cows. They have shown that breed (dairy versus suckling), animal morphology in a breed (selection on body conformation or not), and interaction with finishing diet, can modify the fat deposition in the carcass and meat, the muscle fiber type, the collagen content and composition, and in consequence the meat quality in terms of tenderness, flavor, and juiciness. Moreover, the effects of finishing factors are different according to the animal category (young bulls or cull cows) [11]. Nevertheless, there are few experiments with cull cows because it is difficult to create homogenous experimental groups in terms of body condition score, weight, age, and raising practices before the experimentation (e.g., diets, sanitary events, and compensatory growth).

Moreover, most of the results have been produced in experimental conditions (trials dealing with one or two factors) and are difficult to apply to real multifactorial conditions such as farm management practices [12]. At farm scale, few experiments have studied the effects of rearing practices (diet) on meat quality of *Rectus abdominis* (RA) and *Longissimus thoracis* (LT) [3,13,14]. They have focused their work on the relationships of different types of finishing diets on beef meat quality traits (mainly tenderness) of heifers [3], young bulls [13], and cull beef cows [14], but they did not study factors such as age or animal type and their interaction with finishing diet. For these reasons, our objective was to assess the effects of the age, animal type (suckling ability, morphology) of cull beef cows and the finishing practices (forage and level of concentrate) on meat quality traits of RA (tenderness, color, juiciness, and flavor) and LT (tenderness and color) muscles in relation to their composition (fiber type, enzyme activities, fat content, and collagen content).

This work has been performed on protected denomination of origin (PDO) Maine-Anjou (MA) cull cows. All the animals raised in this PDO belong to the local suckling breed, Rouge des Prés (RdP), which was a dual-purpose breed until the late 1980s. We chose the population of PDO MA cull cows slaughtered in a year (around 1300 cows) for selection of our experimental animals because it is recognized for having the following diverse study factors at farm scale: cull cow morphology (age, body conformation, carcass weight, and fat deposition) and finishing practices [15].

## 2. Materials and Methods

### 2.1. Sampled Animals

Our objective was to sample 10% of the total population of PDO MA cows slaughtered throughout a year, in other words 110–120 cull cows. These cows represented the diverse finishing practices observed in PDO MA farms. A preliminary study on the whole PDO MA farm population conducted by Schmitt et al. [15] identified a representative selection of 45 farms based on their finishing practices (forage type, amount of concentrate, and the finishing period). In 2010, 111 cull cows from these farms and slaughtered throughout the year were selected and sampled.

The cows were collected in a commercial slaughterhouse (Elivia, Le Lion d’Angers, France) following standardized slaughtering procedures (last feed on the day before slaughter, transport duration less than 15 h, maximum 12-h resting period after arrival with available water, stunning procedure with a captive-bolt stunner, and vertical bleeding), as well as standardized chilling and storing procedures (deep muscle temperature of 6–7 °C achieved in 24 h). At 24 h post-mortem, cold carcass weight and fatness scores were collected. The conformation was judged by a trained classifier according to the EUROP classification with three levels per class (+, =, −) (CE1249/2008 regulation). Scores (from 1 to 5) related to the PDO MA agreement and assessing meat color (visual score), intramuscular fat content (IMF) (visual score), and tenderness (palpation of the fifth rib). For each carcass, the fifth rib from the left half of the carcass and the two RA muscles were removed at 24 h post-mortem. The LT muscle was sampled from the fifth rib the same day. Finally, the LT and the two RA muscles were vacuum packaged. The RA muscle was chosen because it is easily sampled, without any economic depletion of the carcass, and because it is more reactive to the variations of rearing practices than LT muscle [16]. In addition, the LT muscle sample was used as a reference for comparison with other studies using LT muscle and with the RA muscle results.

A 3 cm thick steak and samples of 110 g were removed from the LT muscle section and the left RA muscle for further analysis. The steaks were vacuum packaged, chilled for 14 days at 4 °C and then stored at −20 °C until shear force measurements were performed. From the 110 g, 10 g were used for the following fiber characterizations: fiber cross-section area, myosin heavy chains (MyHC) proportions, and enzyme activities. Samples intended for fiber area measurement were cut into 2 duplicates of 2 cm x 3 mm side cubes and stacked on cork in order to position the muscular fibers, progressively frozen in isopentane and then in liquid nitrogen, and then stored at −80 °C until analysis. Samples intended for MyHC proportions and for enzyme activities were cut into small cubes (down to 1 mm side), frozen in liquid nitrogen, and stored at −80 °C. The remaining muscles, 100 g, were used for the IMF, total, and soluble collagen content measurements. The samples were cut into 5 mm side cubes, freeze dried for 72 h and ground to obtain a meat powder. Then, the powder of each sample was vacuum packaged and stored at 4 °C until analysis.

The right RA muscles were vacuum packaged and chilled for 14 days at 4 °C for aging. After aging, they were frozen and stored at −20 °C until sensory analysis.

### 2.2. Physicochemical Measurements

#### 2.2.1. Intermuscular Fat Content and Carcass Composition

The fifth rib was dissected and intermuscular fat, meat, and bones were separated from each other. Each tissue was weighted. Thereby, the fat to meat ratio of the fifth rib (i.e., the intermuscular fat content) and the carcass composition were calculated according to the equations developed by Robelin and Geay (1975) for Salers breed [17]. The equations used were: (i) Muscle in the carcass (kg) = –11.21 + 0.7449 × carcass weight (kg) − 72.52 × adipose tissue in the rib (kg) + 12.2 × muscular tissue in the rib (kg) and (ii) fat in the carcass (kg) = −5.22 + 0.1489 × carcass weight (kg) + 67.48 × adipose tissue in the rib (kg) − 10.39 × muscular tissue in the rib (kg).

#### 2.2.2. Muscle Fiber Cross-Sectional Area

The fiber cross-sectional area was determined by computerized image analysis on 10-μm thick sections cut using a cryotome MICROM HM 500 M at a temperature of −25 °C [18]. The sections were stained with azorubine colorant to define the histological architecture of the muscle and to measure fiber proportion and diameter. Because the fibers did not have the same areas depending on animals and muscles, between 180 and 220 fibers from two different locations in the muscle were used to determine the mean fiber area using computerized image analysis. The surface area of each type of fiber and the mean fiber area were measured using the Visilog software program developed by Meunier et al. [18].

#### 2.2.3. Myosin Heavy Chain Proportions

The MyHC IIx, IIa, and I isoforms were separated according to their molecular weight using the electrophoretic method developed by Picard et al. [19]. Around 100 mg of muscle were ground in 5 mL of a buffer solution containing 0.5 M NaCl, 20 mM Na pyrophosphate, 50 mM tris, 1 mM EDTA and 1 mM dithiothreitol. After centrifugation at 2500 × *g* for 10 min at 4 °C, 500 µL of supernatant were removed and diluted 1:1 v/v with glycerol. The protein content was measured by spectrometry according to Bradford [20]. Thereby, a volume of supernatant containing 5 µg of proteins was analyzed using a sodium dodecyl sulfate polyacrylamide gel electrophoresis (SDS-PAGE) according to Picard et al. [19]. After migration, the gels were stained in a solution of R250 Coomassie blue. The proteins were fixed in a solution made with ethanol (30%) and acetic acid (5%) for 20 min at room temperature. The gels were incubated in a colored solution containing propanol 2 (25%), acetic acid (10%) and Coomassie blue R250 (2 g/L) for 20 min. Then, the proportion (%) of the three MyHC was determined by densitometric analysis using an ImageQuant Software (Amersham Biosciences/GE Healthcare, Uppsala, Sweden).

#### 2.2.4. Enzyme Activities

Muscle samples were homogenized with a polytron in a 5% (wt/v) solution with 10 mM tris (pH 8.0), 0.25 M sucrose, and 2 mM EDTA. One aliquot of homogenate was centrifuged at 10,000 × *g* for 10 min at 4 °C for determination of lactate deshydrogenase (LDH) and isocitrate deshydrogenase (ICDH) activities. The LDH activity was measured by following the disappearance of nicotinamide adenine dinucleotide, reduced form (NADH) at 340 nm, and ICDH activity was measured by following the reduction of nicotinamide adenine dinucleotide phosphate (NADP) at 340 nm. LDH activity was determined according to Ansay [21], using 10 μL of supernatant diluted four-fold, in 2.9 ml of a reaction mixture that contained 50 mM triethanolamine (pH 7.5), 5 mM EDTA, 0.235 mM NADH and 2 mM pyruvate. Pyruvate concentration was determined for maximum LDH activity in bovine muscle. ICDH activity was determined according to Briand et al. [22], using 200 μL of supernatant in 2.7 mL of a reaction mixture that contained 36 mM Na_2_HPO_4_, 0.5 mM MnCl_2_, 0.05% Triton X-100, 0.35 mM NADP and 1.3 mM isocitrate (pH 7.5). All enzyme activities were measured at 25 °C, performed in duplicate, and are expressed as micromoles of substrate concerted per minute and per gram of wet muscle (μmol/min/g).

#### 2.2.5. Intramuscular Fat Content

An Accelerated Solvent Extractor 200 (Dionex Corporation, Sunnyvale, CA, USA) was used to measure out the IMF content of a large number of samples (111 cull cows × 2 muscles × 3 repetitions, *n* = 666). Exactly 1 ± 0.001 g of meat powder was placed in a 22 mL extraction cell previously prepared with a cellulose filter and silicon balls. The IMF was extracted with petroleum ether at a temperature of 125 °C and a pressure of 103 bar. The petroleum ether containing IMF was collected and transferred into an evaporation vial previously weighted (± 0.001 g). After 15 min of evaporation, the vial was placed in a drying oven at 105 °C for 17 h and then weighed (± 0.001 g) to determine the amount of IMF in the meat sample.

#### 2.2.6. Total and Soluble Collagen Contents

The total collagen content of raw meat was derived from the hydroxyproline concentration (collagen = 7.5 × HyPro) determined on five replicates using the method of Bergman and Loxley [23] adapted by Bonnet and Kopp (1984) [24]. The insoluble collagen content was determined following the same procedure on the residue obtained after heating the meat sample in a tris–HCl 0.02M, NaCl 0.23M, pH 7.4 buffer solution (1:5 w/v) at 90 °C for 2 h and subsequently discarding the heat-soluble fraction as described by Bonnet and Kopp [25].

#### 2.2.7. Warner-Bratzler Shear Force

The shear force was measured according to the method developed by Honikel [26] using a Warner-Bratzler shear device (Synergie200 texturometer). After thawing 48 h at 4 °C, the RA and LT steaks were placed for 4 h in a thermostated bath at 18 °C. Then, they were cooked using an Infragrill E (Sofraca, France) set at 300 °C until the temperature at the heart of the steak reached 55 °C. From 3 to 5 test pieces (1 × 1 × 4 cm) were taken from the heart of the steak in the direction of the fibers and 3 to 4 repetitions per test tube were carried out. A 1 kN load cell and a 60 mm/min crosshead speed were used (universal testing machine, MTS, Synergie 200H). The peak load (N) and energy to rupture (J) of the muscle sample were determined.

#### 2.2.8. Color

Meat color was monitored using a portable spectrocolorimeter (Minolta 508i, Minolta Konica, Japan) on LT and RA after a 30-min blooming period (24 h post-mortem, the day of cutting). The illuminant, D65, was the chosen because it closely approximates daylight [27]. The spectrocolorimeter was calibrated before measurement using a standard white calibration tile (Y = 93.58, x = 0.3150, y = 0.3217). Color coordinates were calculated in the CIELAB system: L* (lightness), a* (green to red color components), and b* (blue to yellow color components). Measurements were taken at nine locations on each muscle. Three consecutive measurements were averaged to give one value. Thereby, three values per muscle were obtained.

### 2.3. Sensory Analysis

The right RA muscles were thawed 48 h before sensory analysis at 4 °C. Then, they were cut into 15 mm steaks and cooked for 1 min 45 s in an Infra grill Duo Sofraca set at a temperature of 300 °C (SOFRACA, Morangis, France) to reach a core temperature of 55 °C. After cooking, the steaks were cut into 20 mm cubes that were served on a plastic plate at an internal temperature of 55 °C. Sensory assessment was conducted by a 12-member test panel trained in meat sensory analyses. The panelists evaluated the cooked samples for initial tenderness, overall tenderness, initial juiciness, final juiciness, global flavor, bovine flavor, persistence, and overall appreciation. Initial tenderness was defined as the ease of rupture at the first bite, whereas overall tenderness indicated the ease of rupture along chewing. Initial juiciness was defined as the released juice at the first bite; the final juiciness indicated the released juice at the end of chewing. The persistence characterized the duration of the taste’s persistence all along chewing. Each attribute was rated on a nongraduated scale from 0 to 10 points. At each session, a monadic presentation of 6 RA muscle samples was completed, each sample being selected in random order. The sessions were carried out in a sensory analysis room equipped with individual boxes, under artificial noncolored lighting.

### 2.4. Surveys

For each sampled cow, the rearing practices were recorded using a survey carried out by directly interviewing farmers. The questionnaire included both quantitative and qualitative information about the finishing period and the characteristics of the cow related with its genetic type, its age at slaughter and other indicators of its management. More precisely, the information collected in the survey that related to diet characteristics of the finishing period was:

- The season of the finishing period (seasons are defined as spring (April to June), summer (July to August), autumn (September to November) and winter (December to March));
- The part of hay, either haylage or grass in the finishing diet (% DM of offered forage);
- The amount of concentrate in the diet (kg/day). Concentrate was defined by the amount of raw material (cereals, soya meal, dried beet pulp, …) or balanced compound feed purchased by the farmer;
- The duration of the finishing period (days);
- An estimation of the activity during the finishing period (% finishing days spent outside in a paddock);
- The number of cows in the finishing fattening batch;
- Duration and of the period between the last weaning and the beginning of the finishing period (days).

The information collected in the survey that related to the cull cow characteristics was:

- Birth weight from birth notification (kg);
- Age at first calving (months);
- Age at slaughter (months);
- Parity (i.e., calving number);
- Ascendance characteristics (muscular conformation x muscular conformation, maternal skills x maternal skills, muscular conformation x maternal skills);
- Estimated suckling value by the farmer (i.e., milk production; 0 for no ability to 10 for high ability);
- Sanitary events during productive period (yes/no);
- Long day-open periods (yes/no);
- Potential gestation during the finishing period (yes/no);
- Suckling during the finishing period (yes/no);
- The reason of culling (sanitary/other).

### 2.5. Statistical Analysis

The purpose of our analysis was to create clusters of cull cows based on finishing practices and cull cow morphology, and thereby to compare the created clusters on their muscle composition and meat quality traits. Two multivariate analyses, according to finishing practices and cull cow morphology, were performed. First, two principal component analyses (PCA) were implemented using a ade4 package (acp.dudi procedure) in the R software on the following discriminating and active variables: (i) part of different forages, amount of concentrate, duration of the finishing period, and activity for the finishing clustering; (ii) birth weight, age at first calving, age at slaughter, calving number, estimated suckling value, and carcass weight for the cull cow type clustering. Data were automatically normalized in the analysis in order to give equal weight to each variable in the PCA analysis. The other variables were not used because they were redundant or not discriminant. Afterwards, the item coordinates on the PCA dimensions were included in two hierarchical cluster analyses (HCA, ade4 package, cah procedure based on Ward’s method, and R software). The program identifies the cluster, which has a minor variance within groups and the greater variance between groups. The HCA was done to identify different classes of finishing practices and cull cow types. The classes were reported in the database; each cow was associated with a class of finishing practices and cull cow type.

Classes of finishing practices, of cull cow types and their interactions were compared on data used for PCA, muscle composition, and meat quality traits. Variance analysis and mean multiple comparisons with one factor were carried out using the general linear model (GLM) procedure from SAS. Multiple comparisons of the adjusted means (LSMEANS) were done using the PDIFF option of the GLM procedure.

## 3. Results

### 3.1. Cull Cow and Finishing Practices Clusters

#### 3.1.1. Cull Cow Type Clusters

The PCA distinguished three main components (total proportion of variability explained = 75.2%). The proportions of variability explained were 33.0%, 23.7%, and 18.5% and the eigenvalues were 1.65, 1.19, and 0.92 for the first, second, and third components, respectively. The coordinates of each item on the three PCA components were used to perform the HCA. It led to three clusters which were named Ylight, Omilk, and Yheavy (Table 1). Fifteen cows could not be included in the clustering due to a lack of data for the variables used in the analyses.

The first cluster is composed by light carcasses (415 kg on average) from young cull cows (50 months on average), with a parity below 2 and an average estimated suckling ability (5.5/10) (Ylight, *n* = 51). These cows are not kept for the herd replacement (weak development, low suckling ability) and are quickly culled. The second cluster is composed by heavy carcasses (466 kg on average) from old cows (95 months on average) with a parity above 4 and a high estimated suckling ability (7.7/10) (Omilk, *n* = 32). These cows are kept in the herd because they fit with the following objectives of the farmers: good skeletal and muscular development, reproductive performance, and suckling ability. Finally, the third cluster is composed by heavy carcasses (474 kg on average) from young cows (54 months on average) with a parity below 2 and a low suckling ability (4.4/10) (Yheavy, *n* = 12). These cows have a good muscular development and are quickly culled because of their higher economic value (related to a better carcass yield than the other classes). We observed no difference among clusters in terms of finishing practices.

#### 3.1.2. Finishing Practices Type Clusters

The PCA distinguished three main components (total proportion of variability explained = 74.9%). The proportions of variability explained were 30.5%, 24.7%, and 19.7% and the eigenvalues were 2.14, 1.73, and 1.38 for the first, second, and third components, respectively. The coordinates of each item on the three PCA components were used to perform the HCA. It led to four clusters which were named LongF, HayF, ConcF, and PastF (Table 2). Fourteen cows could not be included in the clustering due to a lack of data for the variables used in the analyses.

The first cluster is characterized by a long finishing period (142 days on average) and a mix of hay and haylage based diet supplemented with 5.8 kg/day of concentrate on average (LongF, *n* = 17). Even if the daily amount of concentrate is the lowest among all the classes, the total amount distributed throughout finishing period is close to the average of the total population (819 kg). The second cluster is characterized by a short finishing period (80 d on average) and a hay-based diet supplemented with 8.0 kg/d (total = 632 kg) of concentrate (HayF, *n* = 41). The third cluster is characterized by duration of the finishing period close to the average of the total population (107 d). The diet is composed by hay supplemented with a high level of concentrate (9.7 kg/d and 1029 kg in total) (ConcF, *n* = 18). Finally, the fourth cluster is characterized by a short finishing period (86 d on average) and a pasture diet supplemented by 7.6 kg/d (total = 665 kg) of concentrate (PastF, *n* = 21). These clusters are consistent with the diverse practices in the PDO MA farm population [15]. We observed no difference among clusters in terms of prefinishing practices (8 weeks between weaning and finishing leading to similar body condition among cows) and cull cow characteristics.

### 3.2. Effect of Cull Cow Clusters on Muscles and Meat Quality Traits

#### 3.2.1. Longissimus Thoracis

There was no difference between clusters on total and insoluble collagen contents and shear force measurements (Table 3). Omilk LT had higher IMF content and fat-to-muscle ratio, in comparison to Ylight and Yheavy LT (+5.8 and +5.2 for IMF content, *p* < 0.01; +7.1 and +6.5 for fat-to-muscle ratio, *p* < 0.01). Yheavy LT tended to have lower cross-sectional area of fibers than Omilk LT (−477 µm², *p* ≤ 0.1). The proportion of MyHC IIa was higher in the LT of Yheavy LT in comparison to Ylight and Omilk LT (+9.5 and +8.8 percentage points (pp), respectively, *p* < 0.05). On the other hand, the proportion of MyHC IIx tended to be lower (–9.5 and –6.8 pp, respectively, *p* ≤ 0.1) and the proportion of MyHC I did not differ between classes. Compared to Omilk LT, the LDH activity in Yheavy LT tended to be lower (−73 µmol/min/g, *p* ≤ 0.1). Finally, the b* index was higher in Yheavy LT in comparison to Ylight and Omilk LT (+1.13 and +0.63, respectively, *p* < 0.05).

#### 3.2.2. Rectus Abdominis

There was no difference between clusters on ICDH and LDH activities, fiber size, total and insoluble collagen contents, shear force, and color (Table 4). In comparison to Ylight RA, Yheavy RA also had lower proportions of MyHC IIx (–7.2 pp, *p* < 0.05), but in favor of the proportion of MyHC I (+6.1 pp, *p* < 0.05). The Yheavy RA tended to have higher IMF content than Ylight RA (+ 4.8 g/100 g, *p* < 0.1). Sensory scores were always below 5/10. The panelists were initially trained for LT analysis, not RA, probably explaining why scores were so low. Initial tenderness tended to be lower in Ylight RA, in comparison to Yheavy and Omilk RA. Overall appreciation tended to be higher in Omilk RA (Table 4).

### 3.3. Effect of Finishing Practices Clusters on Muscles and Meat Quality Traits

#### 3.3.1. Longissimus Thoracis

We observed no effect of finishing practices on ICDH and LDH activities, fiber size, total and insoluble collagen contents, shear force, and IMF content (Table 5). The fat-to-muscle ratio was higher in ConcF LT in comparison to LongF, HayF, and PastF LT (+7.1, +8.8 and +7.2, respectively; *p* < 0.05). The proportion of MyHC IIa in PastF LT, in comparison to LongF, HayF, and ConcF LT, were higher (+9.0 pp, +6.2 pp and +10.4 pp, respectively, *p* < 0.01) and the b* index tended to be higher (+1.2, +0.8, and +0.8 for b* index, respectively, *p* ≤ 0.1). Finally, the a* index was lower in HayF LT in comparison to LongF, ConcF, and PastF LT (–0.8, –0.9, and –0.8, respectively; *p* < 0.01).

#### 3.3.2. Rectus Abdominis

Finishing practices have only influenced the proportion of MyHC I in RA muscle. LongF RA were characterized by a lower proportion of MyHC I in comparison to HayF, ConcF, and PastF RA (–4.6 pp, –5.9 pp and –5.3 pp, respectively, *p* < 0.05) (Table 6). Sensory traits were weakly modified by finishing practices. Initial tenderness was lower in HayF RA in comparison to ConcF RA (–0.4 pp, *p* < 0.05). And juiciness was higher in LongF RA in comparison to HayF, ConcF, and PastF RA (+0.3 pp, +0.3 pp, and +0.3 pp, respectively, *p* < 0.01) (Table 6).

### 3.4. Effect of the Interaction Between Cull Cow Clusters and Finishing Practices Clusters on Muscles and Meat Quality Traits

The effects of the interaction were assessed by considering four finishing practices modalities and only two of the cull cow clusters (Ylight and Omilk). The number of Yheavy cows was too low to include this modality in the statistical analysis. We only observed two trends of interaction effects on IMF content and fat-to-muscle ratio in LT (*p* ≤ 0.1). Finishing practices had no effect on IMF content and fat-to-muscle ratio in LT in Ylight LT. However, in Omilk LT, the IMF content tended to be high when cows were finished according to LongF practices (IMF content = 25.9%, fat-to-muscle ratio = 33.6%); conversely, the fat-to-muscle ratio was high when cows were finished according to ConcF practices (IMF content = 17.3%, fat-to-muscle ratio = 44.2%). These results might suggest that the partition of lipid deposition between inter- and intramuscular fat depend on feeding practices in Omilk cull cows.

## 4. Discussion

In comparison to LT muscle, RA muscle has significantly larger fibers, higher proportion of MyHC I at the expense of MyHC IIa, higher ICDH activity and lower LDH activity, higher shear force, higher collagen and IMF contents, and darker but less red and yellow meat. These differences match the results of [16] and will lead to strong differences in meat quality traits between LT and RA muscles, especially tenderness.

### 4.1. Effect of Cull Cow Clusters on Muscle Characteristics

In our study, the differences in meat quality traits among cull cow clusters are stronger for LT than RA. This result is consistent with Soulat [28] but not Oury et al. [16] who observed stronger differences for RA than LT of Charolais heifers in relation to several finishing practices.

Omilk cows are older, heavier, and have a higher parity and suckling ability than Ylight and Yheavy cows. These cows are kept in the herd for their good maternal skills (milk production to suckle the calves). Moreover, Jurie et al. [5] found no relation between age and carcass weight of suckling cull cows, whereas Liénard et al. [29] observed an increase of carcass weight of suckling cull cows until five to six years of age and then a 10 kg decrease when cows are slaughtered at eight or nine years of age. In our study, at least we can assume that the carcass weight and conformation of Omilk and Yheavy cows are similar and significantly higher than those of Ylight cows.

Omilk LT are characterized by larger fibers, and higher IMF content and fat-to-muscle ratio than Yheavy and Ylight LT. The latter result also means that the proportion of adipose tissue in the carcass is higher for Omilk cows in comparison to Yheavy and Ylight cows (19.4% versus 18.3 and 17.4%, respectively, *p* = 0.003). These differences could be related to the age and the suckling ability of the Omilk cows. Jurie et al. [5] have observed no difference in LT composition (IMF content, MyHC, fiber size and proportion of fat tissue in the carcass between groups of suckling cull cows differing in age (4–5, 6–7 and 8–9 years). In this study, the body composition score at slaughter was similar for all cull cows and could explain why no difference was observed. Moreover, between 4 and 9 years of age (as it was observed for RdP cows in our study), the biological mechanisms involved in fiber size and metabolism modifications do not exist anymore (growth of young cows [5]) or (aging of old cows [5]). The suckling ability could also explain the differences observed between Omilk cows and the others. It is well known that dairy animals (dairy breeds or dairy line within a suckling breed) tend to deposit more fat [5] and to have higher proportions of MyHC I in their muscles [30] when aging. For these reasons, Omilk LT composition would probably be intermediate between that of dairy and suckling cull cows.

Yheavy LT, in comparison to Omilk and Ylight LT, have lower MyHC IIx proportion and higher MyHC IIa proportion and tend to have higher glycolytic activity. Moreover, Yheavy RA, in comparison to Omilk and Ylight RA, have higher MyHC I proportion, and lower MyHC IIx proportion, without any difference in enzyme activities. These cows probably belong to an intra-breed muscular line as farmers are known to select animals on maternal abilities (RDP was a dual-purpose breed until the 90s) or muscular development abilities (some bulls have the double-muscle gene). The animals are leaner, the suckling ability is low, and the LT composition is closer to that of suckling cull cows (Limousine, Charolaise, Belgium Blue), with a higher proportion of IIa and higher glycolytic activities [30,31].

For the two muscles, we observed no difference in total and insoluble collagen contents between cull cow clusters, and in accordance to these results, no difference as well in shear force values. Our results are consistent with previous studies that have shown no relation between age and total and insoluble collagen contents in LT muscle of suckling cull cows [3,4,5]. Gerhardy [12] has also shown similar total collagen content between dairy cull cows (62 months at slaughter on average) and dairy heifers (23 months at slaughter on average). However, they observed a higher insoluble collagen content in the LT of suckling cows, in accordance with Purslow [32]. The existence of a long finishing period (at least 60 days) in our study could explain why there is no difference in insoluble collagen contents between cull cow clusters. During finishing, the collagen frame (perimysium) is remodeled. As the collagen turnover is long (half-life around 45 days), the effect of the remodeling fades after several months [32]. Thus, as finishing lasted more than 45 days in our study, we could assume that the remodeling of the collagen frame has been similar causing similar insoluble collagen contents between cows.

### 4.2. Effect of Finishing Practices Clusters on Muscle Characteristics

In our study, the differences in meat quality traits among and finishing practices clusters are weak for the two studied muscles. The main differences observed, mainly in LT muscle, concerned MyHC fibers proportions and color indices. This shows that RA is less reactive than LT to finishing practices as also stated in the literature [16].

MyHC IIx and IIa proportions are lower in the PastF LT, and the a* and the b* color indices are higher in the PastF LT, as compared with other LT. Muscle pH, physical activity, IMF content and color, and age are the main factors explaining meat color differences [2]. In our study, physical activity, diet, and IMF color are the only factors that could explain our results, other factors being similar between clusters [14]. Priolo et al. [33] have shown that the L* index is lower (related to a darker meat) when cattle graze during finishing. The physical activity and grazing (in comparison to hay diet), by modifying the fiber metabolic activity (mainly oxidative) [34], would lead to higher myoglobin content in the muscle and thereby the higher L* index [35]. However, at least 100 days of grazing would be necessary to observe significant effects [33] and probably would be adequate to increase the a* index. On the other hand, Kerth et al. [8] have shown that the b* index of subcutaneous fat is higher when cattle graze during finishing. Assuming that the IMF b* index is also higher, this could explain the higher b* index in PastF LT [36].

In ConcF LT, the fat-to-muscle ratio was higher without any effect on IMF content, whereas, in LongF LT, the IMF content was higher without any effect on the fat-to-muscle ratio. Even if the body condition is not known at the beginning of the finishing period, it seems that the finishing duration and the amount of energy concentrates (per day) influence the allotment of adipose tissue deposit during finishing [11]. A short finishing period with a high proportion of concentrate in the diet, as observed in the ConcF cluster, would lead to a deposit of adipose tissue in either the internal or intermuscular fat [3]. As shown by Robelin et al. [1], first the fat deposit occurs in the subcutaneous and internal fats, then in the intermuscular fat, and finally in the IMF. A short finishing period with a low forage-to-concentrate ratio diet promotes fat deposition as subcutaneous and internal fat, whereas a long finishing period with a high forage-to-concentrate ratio diet deposits fat in the IMF. Thereby, these feeding practices influence the IMF content and the fat composition of the carcass [9,37].

Shear force was not different among finishing practices clusters. This is consistent with the extensive literature that shows no or very few effects of forage type and amount of concentrate during finishing on shear force [3,36,38]. Nevertheless, the tenderness assessed by sensory analysis tended to be higher in ConcF and PastF RA, in comparison to HayF and LongF RA. Even if the main factors influencing tenderness (total and insoluble collagen contents, IMF content, carcass conformation, and fibers) were similar between clusters, the finishing practices could explain this slight effect. Vestergaard et al. [35] have shown that a low forage-to-concentrate ratio in the finishing diet, as observed in ConcF, could improve meat tenderness. Jurie et al. [34] have shown that a finishing diet based on grazing, as observed in PastF, could improve meat tenderness by modifying the metabolic activity and fiber types in the muscles.

In our study, cull cows were selected and collected in farms. Their characteristics before the finishing period (body condition score) and their history (growth, reproductive performance, diets, fat lipomobilization, sanitary events, …) were highly variable. Although this information was collected (survey), its variability was too high to take it into consideration in our statistical analysis (clustering). This could have interfered in our analyses. Indeed, Apple et al. [39] have observed a linear relationship between the body condition score and the fat composition in the carcass of cull cows. Furthermore, it is well known in younger animals (heifers and steers) that the characteristics of the growth period (compensatory growth for instance) impact the effects of the finishing periods on the carcass composition and the meat quality (subcutaneous fat, IMF content, and tenderness) [40,41]. For that reason, often the growth period and the body condition scores are considered when animals are allocated to the experimental treatments in trials dealing with the effect of finishing practices on meat quality of heifers and steers. Thus, it could be interesting to perform another experiment taking into account those factors in order to study the effect of the interaction between the history of the cow before culling and the finishing practices on carcass and meat quality traits.

By exploring the differences between muscular and dairy lines within a local breed, our study gives new insights into the effect of animal type on meat quality. Our study is original because it considered the interaction between animal type and finishing practices at farm scale on the meat quality of cull beef cows. Finishing practices have less effect than animal type on RA and LT meat properties. Their effects also differed according to muscle type (RA or LT) and cull cows types on muscle composition. The effects observed on meat quality are directly related to farmers’ practices and provide new advice and modifications in culled cows finishing practices to improve meat quality. As we only performed sensory analyses on RA muscle, we can only suppose that those differences might have effects on sensory attributes among muscles. It could be interesting to study other muscles to assess whether the effects of the animal type and finishing practices are similar regardless of the muscles considered. Moreover, to enhance characterization of animal types, it could be interesting to create animal clusters including genetic indices (e.g., suckling ability index) as a replacement for qualitative information from farmer surveys. It could also be interesting to increase the number of animals using a multifactorial approach, to study the effect of the overall farm management of cows on carcass and meat quality traits. This means studying the interaction between the practices before culling (sanitary problems, compensatory growth, feeding system, etc.), the animal characteristics at the beginning of the finishing period (e.g., body condition score), the animal type (as observed in our study) and the finishing practices. This interaction could partially explain the lack of effects observed among clusters (IMF content for instance among finishing clusters).

## Figures and Tables

**Table 1:** Characteristics of the cull cow clusters identified by the hierarchical cluster analysis (adjusted mean from anova ± standard error of the mean).

	Ylight	Omilk	Yheavy	*p*
N	51	32	13	
Cold carcass weight, kg	415 ^a^ ± 3.0	466 ^b^ ± 5.9	474 ^b^ ± 4.0	***
Estimated suckling value, /10	5.5 ^b^ ± 0.17	7.1 ^c^ ± 0.14	4.4 ^a^ ± 0.26	***
Parity	1.5 ^a^ ± 0.09	5.2 ^b^ ± 0.23	1.7 ^a^ ± 0.26	***
Slaughter age, months	50.4 ^a^ ± 1.41	95.3 ^b^ ± 3.07	54.0 ^a^ ± 2.99	***
Age at 1st calving, months	32.8 ^a^ ± 0.53	32.0 ^a^ ± 0.84	33.5 ^b^ ± 0.84	***

***: *p* < 0.001; Ylight = cluster composed by young and light cows with medium suckling value; Omilk = old and heavy cows with good suckling value; Yheavy = young and heavy cows with low suckling value; ^a,b,c^ values within a row with different superscripts differ significantly at *p* < 0.05.

**Table 2:** Characteristics of the finishing practices clusters identified by the hierarchical cluster analysis (adjusted mean from anova ± standard error of the mean).

	LongF	HayF	ConcF	PastF	*p*
Nb	17	41	18	21	
Duration, days	142 ^c^ ± 6.3	80 ^a^ ± 1.9	106 ^b^ ± 3.2	86 ^a^ ± 6.0	***
% finishing days spent outside	23 ^a^ ± 8.0	47 ^b^ ± 7.8	40 ^b^ ± 10.1	94 ^c^ ± 4.9	***
Grazed grass, % forage DM	8 ^a^ ± 2.7	5 ^a^ ± 1.5	4 ^a^ ± 1.7	83 ^b^ ± 2.7	***
Haylage, % forage DM	46 ^b^ ± 9.8	39 ^b^ ± 6.6	16 ^a^ ± 5.7	2 ^a^ ± 1.2	***
Hay, % forage DM	46 ^b^ ± 8.6	55 ^b^ ± 6.4	80 ^c^ ± 5.5	14 ^a^ ± 2.5	***
Concentrate, kg/d	5.8 ^a^ ± 0.31	8.0 ^b^ ± 0.26	9.7 ^c^ ± 0.28	7.6 ^b^ ± 0.57	***
Concentrate throughout finishing, kg	819 ^b^ ± 58.1	632 ^a^ ± 20.0	1029 ^c^ ± 36.5	665 ^a^ ± 68.0	***

***: *p* < 0.001; LongF = long finishing period with a mix hay-haylage diet supplemented with a low amount of concentrate; HayF = short finishing period with a hay or haylage diet supplemented with a medium amount of concentrate; ConcF = medium finishing period with a hay diet supplemented with a high amount of concentrate; PastF = short finishing period with a grazed grass diet supplemented with a medium amount of concentrate; ^a,b,c^ values within a row with different superscripts differ significantly at *p* < 0.05.

**Table 3:** Effects of the cull cow clusters on *Longissimus thoracis* characteristics (adjusted mean from anova ± standard error of the mean).

	Ylight	Omilk	Yheavy	*p*
Nb	51	32	13	
ICDH ^1^	1.06 ± 0.05	1.00 ± 0.06	1.12 ± 0.07	NS
LDH ^1^	709 ^a^ ± 15.2	691 ^a^ ± 17.6	764 ^b^ ± 32.0	†
Fiber size, µm^2^	2878 ^a,b^ ± 94.4	3142 ^b^ ± 126.9	2665 ^a^ ± 102.3	†
IIx, %	15.3 ^b^ ± 2.27	12.3 ^a,b^ ± 2.02	5.8 ^a^ ± 3.10	†
IIa, %	54.3 ^a^ ± 1.85	55.0 ^a^ ± 2.26	63.8 ^b^ ± 3.15	*
I, %	30.4 ± 0.99	32.6 ± 1.66	30.4 ± 1.01	NS
Total collagen ^2^	3.09 ± 0.06	3.09 ± 0.08	2.95 ± 0.12	NS
Insoluble collagen ^2^	2.43 ± 0.05	2.47 ± 0.06	2.26 ± 0.08	NS
Shear force, N/cm^2^	45.6 ± 1.92	45.3 ± 1.69	42.2 ± 2.28	NS
IMF^3^, g/100g DM	15.2 ^a^ ± 0.87	20.0 ^b^ ± 1.14	15.8 ^a^ ± 1.11	**
Fat-to-muscle ratio	29.2 ^a^ ± 1.23	36.3 ^b^ ± 2.31	29.8 ^a^ ± 1.38	**
L*	39.5 ± 0.31	39.9 ± 0.37	39.2 ± 0.83	NS
a*	8.61 ± 0.16	8.79 ± 0.19	9.19 ± 0.33	NS
b*	7.07 ^a^ ± 0.16	7.57 ^a^ ± 0.27	8.20 ^b^ ± 0.29	*

**: *p* < 0.01; *: *p* < 0.05; †: *p* ≤ 0.1; NS: nonsignificant; ^1^ isocitrate dehydrogenase (ICDH) and lactate dehydrogenase (LDH) activities, in µmole/min/g; ^2^ in µg OH-proline/mg dry matter; ^3^ intramuscular fat content; ^a,b,c^ values within a row with different superscripts differ significantly at *p* < 0.05.

**Table 4:** Effects of the cull cows clusters on *Rectus abdominis* characteristics (adjusted mean from anova ± standard error of the mean).

	Ylight	Omilk	Yheavy	*p*
Nb	51	32	13	
ICDH ^1^	1.25 ± 0.5	1.37 ± 0.05	1.35 ± 0.13	NS
LDH ^1^	645 ± 15.0	612 ± 13.9	669 ± 37.6	NS
Fiber size, µm²	3516 ± 118.0	3664 ± 170.3	3743 ± 232.9	NS
IIx, %	27.4 ^b^ ± 1.68	21.3 ^a^ ± 1.78	20.2 ^a^ ± 3.89	*
IIa, %	36.2 ± 1.18	40.0 ± 1.54	37.2 ± 2.84	NS
I, %	36.4 ^a^ ± 1.03	38.7 ^a,b^ ± 1.25	42.5 ^b^ ± 1.78	*
Total collagen ^2^	3.64 ± 0.10	3.50 ± 0.08	3.51 ± 0.17	NS
Insoluble collagen ^2^	2.84 ± 0.08	2.75 ± 0.07	2.70 ± 0.13	NS
Shear force, N/cm²	49.8 ± 1.21	50.6 ± 1.69	49.2 ± 2.61	NS
IMF^3^, g/100g DM	17.3 ^a^ ± 1.07	19.6 ^a,b^ ± 1.15	22.1 ^b^ ± 2.59	†
L*	38.9 ± 0.31	38.7 ± 0.35	38.4 ± 0.67	NS
a*	5.77 ± 0.15	5.88 ± 0.17	5.63 ± 0.23	NS
b*	4.50 ± 0.13	4.78 ± 0.18	4.44 ± 0.31	NS
Initial tenderness, /10	4.7 ^a^ ± 0.06	4.8 ^b^ ± 0.08	4.9 ^b^ ± 0.08	†
Initial juiciness, /10	4.2 ± 0.06	4.2 ± 0.07	4.2 ± 0.07	NS
Global flavour, /10	4.7 ± 0.04	4.8 ± 0.05	4.8 ± 0.09	NS
Persistance, /10	4.5 ± 0.04	4.5 ± 0.05	4.5 ± 0.06	NS
Overall appreciation, /10	2.9 ^a^ ± 0.08	3.2 ^b^ ± 0.11	2.9 ^a^ ± 0.14	†

*: *p* < 0.05; †: *p* ≤ 0.1; NS: nonsignificant; ^1^ isocitrate dehydrogenase (ICDH) and lactate dehydrogenase (LDH) activities, in µmole/min/g; ^2^ in µg OH-proline/mg dry matter; ^3^ intramuscular fat content; ^a,b,c^ values within a row with different superscripts differ significantly at *p* < 0.05.

**Table 5:** Effects of the finishing practices clusters on *Longissimus thoracis* characteristics (adjusted mean from anova ± standard error of the mean).

	LongF	HayF	ConcF	PastF	*p*
Nb	17	41	18	21	
ICDH ^1^	1.06 ± 0.12	1.06 ± 0.05	1.03 ± 0.06	1.02 ± 0.04	NS
LDH ^1^	710 ± 27.5	698 ± 18.5	705 ± 22.8	706 ± 22.9	NS
Fiber size, µm²	2933 ± 184.5	2808 ± 94.6	2986 ± 130.7	2991 ± 160.9	NS
IIx, %	14.5 ± 2.96	12.8 ± 2.60	14.6 ± 2.97	7.0 ± 2.53	NS
IIa, %	53.1 ^a^ ± 2.54	55.9 ^a^ ± 1.97	51.7 ^a^ ± 2.63	62.1 ^b^ ± 3.23	*
I, %	32.4 ± 1.78	31.2 ± 1.05	33.6 ± 1.24	30.9 ± 2.24	NS
Total collagen ^2^	3.06 ± 0.11	3.09 ± 0.06	3.12 ± 0.11	3.14 ± 0.09	NS
Insoluble collagen ^2^	2.43 ± 0.09	2.45 ± 0.05	2.47 ± 0.08	2.47 ± 0.07	NS
Shear force, N/cm²	49.1 ± 3.61	44.2 ± 1.60	43.8 ± 2.91	42.9 ± 1.64	NS
IMF^3^, g/100g DM	18.2 ± 2.03	16.1 ± 0.90	15.8 ± 1.15	15.7 ± 1.17	NS
Fat-to-muscle ratio	30.9 ^a^ ± 2.40	29.2 ^a^ ± 1.21	38.0 ^b^ ± 3.62	30.8 ^a^ ± 1.41	*
L*	40.0 ± 0.55	39.9 ± 0.41	39.6 ± 0.34	39.6 ± 0.53	NS
a*	9.2 ^b^ ± 0.35	8.4 ^a^ ± 0.16	9.3 ^b^ ± 0.26	9.2 ^b^ ± 0.21	**
b*	7.0 ^a^ ± 0.36	7.4 ^a^ ± 0.23	7.4 ^a^ ± 0.26	8.2 ^b^ ± 0.33	†

**: *p* < 0.01; *: *p* < 0.05; †: *p* ≤ 0.1; NS: nonsignificant; ^1^ isocitrate dehydrogenase (ICDH) and lactate dehydrogenase (LDH) activities, in µmole/min/g; ^2^ in µg OH-proline/mg dry matter; ^3^ intramuscular fat content; ^a,b,c^ values within a row with different superscripts differ significantly at *p* < 0.05.

**Table 6:** Effects of the finishing practices clusters on *Rectus abdominis* characteristics (adjusted mean from anova ± standard error of the mean).

	LongF	HayF	ConcF	PastF	*p*
Nb	17	41	18	21	
ICDH ^1^	1.25 ± 0.09	1.29 ± 0.05	1.42 ± 1.00	1.22 ± 0.06	NS
LDH ^1^	633 ± 17.2	656 ± 17.8	616 ± 20.1	648 ± 25.4	NS
Fiber size, µm²	3855 ± 206.6	3466 ± 104.7	3559 ± 195.6	3636 ± 214.9	NS
IIx, %	25.2 ± 2.32	25.5 ± 2.04	24.8 ± 2.34	23.2 ± 2.58	NS
IIa, %	40.8 ± 1.91	36.9 ± 1.36	35.4 ± 2.02	37.6 ± 2.02	NS
I, %	33.9 ^a^ ± 1.46	37.5 ^b^ ± 1.03	39.8 ^b^ ± 1.79	39.2 ^b^ ± 1.52	*
Total collagen ^2^	3.51 ± 0.11	3.67 ± 0.11	3.33 ± 0.09	3.57 ± 0.16	NS
Insoluble collagen ^2^	2.73 ± 0.11	2.87 ± 0.09	2.55 ± 0.07	2.81 ± 0.13	NS
Shear force, N/cm²	55.9 ± 4.69	51.4 ± 1.53	48.7 ± 1.45	50.7 ± 2.34	NS
IMF^3^, g/100g DM	19.8 ± 1.62	17.5 ± 1.07	17.7 ± 1.61	19.8 ± 2.02	NS
L*	39.8 ± 0.36	39.0 ± 0.38	38.8 ± 0.50	38.3 ± 0.35	NS
a*	6.1 ± 0.27	5.5 ± 0.16	5.8 ± 0.26	5.9 ± 0.23	NS
b*	4.5 ± 0.24	4.4 ± 0.16	4.5 ± 0.27	4.6 ± 0.22	NS
Initial tenderness, /10	4.6 ^a,b^ ± 0.11	4.5 ^a^ ± 0.08	4.9 ^b^ ± 0.07	4.8 ^a,b^ ± 0.09	*
Initial juiciness, /10	4.5 ^b^ ± 0.10	4.2 ^a^ ± 0.05	4.2 ^a^ ± 0.10	4.2 ^a^ ± 0.08	**
Global flavour, /10	4.9 ± 0.06	4.7 ± 0.04	4.7 ± 0.05	4.7 ± 0.07	NS
Persistance, /10	4.6 ± 0.06	4.5 ± 0.05	4.4 ± 0.05	4.5 ± 0.07	NS
Overall appreciation, /10	3.1 ± 0.14	2.9 ± 0.08	3.1 ± 0.11	2.9 ± 0.12	NS

**: *p* < 0.01; *: *p* < 0.05; †: *p* ≤ 0.1; NS: nonsignificant; ^1^ isocitrate dehydrogenase (ICDH) and lactate dehydrogenase (LDH) activities, in µmole/min/g; ^2^ in µg OH-proline/mg dry matter; ^3^ intramuscular fat content; ^a,b,c^ values within a row with different superscripts differ significantly at *p* < 0.05.
